# Purification and In Situ Immobilization of Papain with Aqueous Two-Phase System

**DOI:** 10.1371/journal.pone.0015168

**Published:** 2010-12-13

**Authors:** Mingliang Li, Erzheng Su, Pengyong You, Xiangyu Gong, Ming Sun, Diansheng Xu, Dongzhi Wei

**Affiliations:** State Key Laboratory of Bioreactor Engineering, New World Institute of Biotechnology, East China University of Science and Technology, Shanghai, China; University of South Florida College of Medicine, United States of America

## Abstract

Papain was purified from spray-dried *Carica papaya* latex using aqueous two-phase system (ATPS). Then it was recovered from PEG phase by in situ immobilization or preparing cross-linked enzyme aggregates (CLEAs). The Plackett-Burman design and the central composite design (CCD) together with the response surface methodology (RSM) were used to optimize the APTS processes. The highly purified papain (96–100%) was achieved under the optimized conditions: 40% (w/w) 15 mg/ml enzyme solution, 14.33–17.65% (w/w) PEG 6000, 14.27–14.42% (w/w) NaH_2_PO_4_/K_2_HPO_4_ and pH 5.77–6.30 at 20°C. An in situ enzyme immobilization approach, carried out by directly dispersing aminated supports and chitosan beads into the PEG phase, was investigated to recover papain, in which a high immobilization yield (>90%) and activity recovery (>40%) was obtained. Moreover, CLEAs were successfully used in recovering papain from PEG phase with a hydrolytic activity hundreds times higher than the carrier-bound immobilized papain.

## Introduction

Papain (EC 3.4.22.2) is one of the minor constituents (5–8%) in the cysteine endopeptidases extracted from the latex of *Carica papaya* [1]. It is one of the most exploited plant proteases, which has been used in brewing, baking, meat tenderizing, wounds defibrinating, edemas treating, wool anti-shrinking, cells isolating and Fab fragments preparing, etc. [2]. Papain has also been successfully applied in synthesis of many compounds such as peptides, lipoamino acid-based surfactants, esters of amino acids and carbohydrate derivatives [3].

Papain is extracted from the latex of *Carica papaya* fruit. Previously, the commercially available latex, which was seriously contaminated and contained substantial quantities of insoluble material, was usually dried by sun or oven without further purification. Now, the spray-dried latex available in the market is more refined and free from insoluble material [1], [4]. Traditionally, both types of papaya latex are used to purify papain by multi-steps salt precipitation followed by crystallization. However, the process is time-consuming and the purified enzyme still contaminated with other proteases [4], [5]. Another purification strategy which involves various chromatographic techniques including ion exchange, covalent or affinity chromatography, is difficult to scale up and the cost is high [6], [7].

It is important to develop industry-desired procedures which are not only time saving with low cost, but also generate enzyme with high yields and purity. Aqueous two-phase system (ATPS) is such a powerful method which has been extensively exploited to separate or purify biological products from different sources, and generates robust, easy to scale and biocompatible extraction processes [8]. This purification process integrates the clarification, concentration and purification in one unit operation. ATPS forms when two incompatible hydrophilic polymers or a polymer and a salt are mixed in aqueous solution above a critical concentration. Biological products such as enzymes can then be partitioned between the phases and purified to a good extent [9]. Some successful applications of ATPS on large/industrial scale have been demonstrated [10], [11]. In 1990, Kuboi et al. used the ATPS for the separation of papain from papaya latex [12]. Their study showed that the separated papain was still contaminated with chymopapain. In 2006, Nitsawang et al. reported the use of polyethylene glycol (PEG)-(NH_4_)_2_SO_4_ system for purifying papain from fresh papaya latex collected from the papaya fruit directly (which was not commercially available and difficult to handle) [7]. But this study was based on single-factor experimental design, and did not systematically optimize the ATPS process. Furthermore, the study didn't mention how to recover the purified papain from the PEG phase.

An ideal partition of proteins in ATPS can be accomplished by manipulating a variety of system parameters [13]. So it is very crucial to optimize the parameters of ATPS process in purifying papain from papaya latex. Response surface methodology (RSM), which includes experimental design, model fitting, validation and condition optimization, has eliminated the drawbacks of single-factor experimental design and been proved to be powerful and useful for the optimization of ATPS [14], [15].

ATPS extraction of protein mixtures leads to one or several protein fractions, which also contain mainly one of the phase-forming polymers. So another problem for ATPS industrialization is how to recover the target protein from the phase forming polymer. Traditionally, a number of methods can be used for this purpose, such as gel chromatography, ultrafiltration, ion-exchange chromatography and back extraction [16]. However, these methods are complicated, expensive and difficult to scale up. Alternatively, an in situ immobilization method, which is carried out by direct immobilization of the enzyme from the PEG phase onto a support, may be a feasible choice. It avoids the use of other purification steps and can get immobilized biocatalyst at the same time. More importantly, the PEG phase or salt phase can be recycled. Several works had reported this method for the isolation and immobilization of enzymes, and good results had been attained [17]–[19]. In the present work, we optimized the ATPS to purify papain from commercially available papaya latex using RSM. Then the in situ immobilization method was investigated to recover and immobilize the papain from the PEG phase. In addition, preparing cross-linked enzyme aggregates (CLEAs) was preliminarily proposed by Kallenberg et al. as a potential method to recover enzyme from ATPS in a review [20], which inspired us to explore the feasibility of preparing CLEAs from the PEG phase for the first time.

## Materials and Methods

### 2.1. Materials

Spray-dried papaya latex (Papain powder PSM 500) was purchased from ENZYBEL Intl.s.a. (Belgium). PEG 4000 and 6000 were purchased from DingGuo Biotech. Co., Ltd (Shanghai, China). N-α-benzoyl-DL-arginine-*p*-nitroanilide (DL-BAPNA) was purchased from Acros Organics (USA). 2×crystallized papain (Cat.#P4762) was purchased from Sigma-Aldrich (USA). Glutaraldehyde solution (25%) was purchased from Sinopharm Chemical Reagent Co., Ltd. (Shanghai, China). Chitosan (degree of deacetylation ≥95%) was purchased from Golden-Shell Biochemical Co., Ltd. (Zhejiang, China). Immobilization supports: ZH-HA was supplied by GeneRad Biotech laboratory limited (Hong Kong, China). LH-HA was provided by Shanghai Bairui Biotech. Co., Ltd. (Shanghai, China). BB-A was presented from Bik Chemical Technologies Ltd. (Tianjin, China). They were all aminated-acrylic resin. All other chemicals and reagents were obtained commercially and were of analytical grade.

### 2.2. Sample preparation

45 g spray-dried latex powders were dissolved in 250 ml 20 mM-cysteine buffer (containing 1 mM-EDTA, pH 5.7) at 4°C. The resulting suspension was submitted to centrifugation (20,000×g, 4°C, 15 min). The supernatant (approximate 45 mg/ml) used as the starting enzyme solution for ATPS was diluted to different protein concentration.

### 2.3. Aqueous two-phase systems preparation

Aqueous two-phase systems were prepared in a graduated tube with 4 g enzyme solution plus various amounts of PEG (4000 or 6000), salt solution (40% w/w phosphate or 40% w/w (NH4)2SO4) and deionized water to reach a total weight of 10 g. Phosphate solution was prepared using K_2_HPO_4_ and NaH_2_PO_4_, as they display greater solubility than their respective monobasic and dibasic salts [21]. To achieve a certain pH value, different ratios of 40% (w/w) monobasic and dibasic salt solutions were mixed. The pH of enzyme solutions was adjusted with 6 M HCl or NaOH. All system components were thoroughly mixed in orbital shakers at 4 or 20°C for 2 h. To ensure complete phase separation, the systems were centrifuged at 10,000×g for 15 min at respective temperature.

Phase volumes were measured, and then aliquots of the phases were taken to determinate protein concentration and activity. The presence of papain was verified by Basic Protein Native-PAGE and FPLC. Phase composition was determined using phase diagrams reported by Albertsson [22].

### 2.4. Experimental design for ATPS process

#### 2.4.1. Screening of important factors

To achieve the screening of important factors, a Plackett-Burman (P-B) design was adopted. The P-B design is an efficient way to screen the important factors among a large number of variables which are studied at two widely spaced levels (the low level (-1) and high level (+1)). Table 1 showed the design matrix covering seven variables to evaluate their effects and two dummy variables.

**Table 1:** Plackett-Burman design matrix with papain purity and activity recovery.

Run	Variables									Papain purity* (%)	Activity recovery** (%)
	A	B	C	D	E	F	G	H	I		
1	1	−1	1	−1	−1	−1	1	1	1	48.23±1.0	10.02±0.1
2	1	1	−1	1	−1	−1	−1	1	1	56.74±0.6	20.14±0.4
3	−1	1	1	−1	1	−1	−1	−1	1	87.81±1.8	10.68±0.2
4	1	−1	1	1	−1	1	−1	−1	−1	83.50±0.8	15.73±0.2
5	1	1	−1	1	1	−1	1	−1	−1	96.52±1.9	1.54±0.05
6	1	1	1	−1	1	1	−1	1	−1	98.33±1.0	14.77±0.3
7	−1	1	1	1	−1	1	1	−1	1	93.74±1.9	1.84±0.1
8	−1	−1	1	1	1	−1	1	1	−1	96.58±1.9	1.73±0.05
9	−1	−1	−1	1	1	1	−1	1	1	93.40±0.9	15.83±0.5
10	1	−1	−1	−1	1	1	1	−1	1	82.69±1.7	10.04±0.2
11	−1	1	−1	−1	−1	1	1	1	−1	43.22±1.3	10.09±0.1
12	−1	−1	−1	−1	−1	−1	−1	−1	−1	37.24±1.1	25.34±1.0

A: initial protein concentration at a low level (−1) of 10 mg/ml and a high level (+1) of 15 mg/ml; B: PEG molecular weight at a low level (−1) of 4000 Da and a high level (+1) of 6000 Da; C: PEG concentration at a low level (−1) of 10% (w/w) and a high level (+1) of 16% (w/w); D: phase forming salt at a low level (−1) of (NH_4_)_2_SO_4_ and a high level (+1) of NaH_2_PO_4_/K_2_HPO_4_; E: salt concentration at a low level (−1) of 10% (w/w) and a high level (+1) of 14% (w/w); F: temperature at a low level (−1) of 4°C and a high level (+1) of 20°C; G: pH at a low level (−1) of 6 and a high level (+1) of 8; H and I represent dummy variables.

*Determined by FPLC.

**Calculated by total activity of PEG phase/total activity of enzyme solution.

#### 2.4.2. Optimization of screened components

Central composite design (CCD) was employed for determining the optimal conditions of the three most significant factors identified by P-B design. Each variable was designed at five levels with six star points and six replicates at the centre points. 20 experiments were required for this procedure. The alpha value was set as 2. Table 2 showed the CCD design matrix and responses for the papain purity and total activity of PEG phase.

**Table 2:** Design matrix for optimization of papain purity and total activity using CCD.

Run	C_PEG_ (%, w/w)	C_salt_ (%, w/w)	pH	Papain purity* (%)	Total activity of PEG phase (nkat)
1	0 (16)	−2 (10)	0 (6)	92.05±1.8	18.57±0.2
2	1 (18)	1 (16)	1 (7)	97.45±1.9	2.94±0.1
3	−1 (14)	1 (16)	−1 (5)	92.33±0.9	20.67±0.2
4	1 (18)	−1 (12)	1 (7)	97.42±1.0	12.30±0.2
5	1 (18)	−1 (12)	−1 (5)	89.42±0.9	22.74±0.7
6	−1 (14)	1 (16)	1 (7)	97.86±2.0	8.24±0.2
7	0 (16)	0 (14)	0 (6)	98.11±1.0	18.41±0.2
8	0 (16)	2 (18)	0 (6)	100.00±1.0	3.32±0.1
9	0 (16)	0 (14)	2 (8)	96.75±1.9	1.27±0.1
10	−1 (14)	−1 (12)	−1 (5)	87.59±1.8	23.10±0.5
11	0 (16)	0 (14)	−2 (4)	82.85±0.8	24.62±0.7
12	0 (16)	0 (14)	0 (6)	96.01±1.0	17.24±0.2
13	0 (16)	0 (14)	0 (6)	98.64±1.0	16.46±0.3
14	−2 (12)	0 (14)	0 (6)	96.35±1.9	18.95±0.2
15	2 (20)	0 (14)	0 (6)	97.95±0.5	12.13±0.1
16	0 (16)	0 (14)	0 (6)	98.14±1.0	18.16±0.7
17	−1 (14)	−1 (12)	1 (7)	96.16±0.5	13.36±0.1
18	1 (18)	1 (16)	−1 (5)	96.22±1.0	17.77±0.2
19	0 (16)	0 (14)	0 (6)	96.45±1.9	17.53±0.2
20	0 (16)	0 (14)	0 (6)	97.53±1.0	17.72±0.1

Actual variables are given in parentheses.

*Determined by FPLC.

Statistical design and analysis were performed using design expert software (version 7.1.6, Stat-Ease, Inc., Minneapolis, MN, USA).

### 2.5. Determination of protein content

The protein content in the samples was determined by Bradford method using bovine serum albumin (BSA) as standard [23].

### 2.6. Enzyme assay for amidase activity

The amidase activity of the samples was measured using DL-BAPNA as substrate [24], [25]. Substrate stock solution was 10 mM DL-BAPNA in DMSO. The activity buffer (pH 6.8) contained citrate-borate-phosphate (100 mM each), 2.5 mM DTT and 1 mM EDTA.

*x* ml of enzyme solution was incubated with (1.8-*x*) ml activity buffer at 37°C for 15 min. Then 0.2 ml substrate preincubated at 37°C was added to start the reaction. After 15 min, the reaction was stopped with 0.5 ml of 50% acetic acid. When the immobilized enzymes or CLEAs were used, *x* g of the enzyme was incubated with 1.8 ml activity buffer. The release of *p*-nitroaniline was determined spectrophotometrically at 410 nm using a ε410 = 8800 M^−1^cm^−1^. *x* was chosen so that ΔA_410_ never exceeded 1.0 after 15 min [26], [27]. One unit of activity (nkat) is the amount of proteinase (free, immobilized or CLEAs) that hydrolyses one nmol of substrate per second under the abovementioned conditions.

### 2.7. Purity analysis by fast protein liquid chromatography (FPLC)

Purity of the purified papain was evaluated by ion-exchange chromatography on FPLC (AKTA Explorer 100, Amersham Biosciences, Uppsala, Sweden). Chromatographic studies were achieved on a HiTrap™ SP-FF (1 ml) column. All the top PEG phase samples were diluted to 1 mg/ml for the FPLC.

The mobile phase A of 50 mM NaAc buffer (pH 5.0) and the mobile phase B of 50 mM NaAc 1 M NaCl (pH 5.0) were used for FPLC. The mobile phases were filtered prior to use. The sample (1 ml) was loaded onto the column pre-equilibrated with phase A, and the chromatographic separation was carried out using a gradient (5–50 Column Volume, phase B from 0% to 70% and 50–60 Column Volume, keep at 70% phase B) at the flow rate of 1.0 ml/min. The UV-900 detector was set at 280 nm for measuring the protein's aromatic residues. The elution peak of papain was confirmed by standard papain and referring to the published works [24], [28]. The peak areas of papain and other proteins were obtained from an automatic integrator. The purity of papain was specified as the percentage peak area of papain with respect to the total peak area.

### 2.8. Basic protein native-polyacrylamide gel electrophoresis (PAGE)

The experiment was carried out according to the method of Reisfeld et al. [29] and Dekeyser et al. [25] with some modification. The stacking gel consisted of 5% polyacrylamide (pH 6.8), and the resolving gel consisted of 15% polyacrylamide (pH 4.3). The electrode buffers in upper and lower chambers consisted of 0.35 M β-alanine-0.14 M acetic acid (pH 4.5). The protein sample was diluted (1∶1, v/v) prior to loading onto the gel with loading buffer containing 25% stacking buffer, 20% glycerol and 0.004% basic fuchsin (used as a tracking dye). Electrophoresis was run at a constant current of 30 mA at 4°C. The protein samples migrated towards the cathode during electrophoresis. The gel was stained with 0.1% Coomassie Blue R250.

### 2.9. In situ immobilization of papain from PEG phase

In the ATPS, the papain was enriched in the top PEG phase and still mixed with PEG. Therefore, it is important to further recover papain from the PEG phase. An “in situ” enzyme immobilization method, which contained carrier-bound and carrier-free immobilization (CLEAs), was assessed for this purpose in this work. The ATPS used here was consisted of 40% (w/w) 15 mg/ml enzyme solution, 14.33% (w/w) PEG 6000, 14.27% (w/w) NaH2PO4/K2HPO4 and pH 5.77 at 20°C.

#### 2.9.1. Activation of aminated supports

The supports (ZH-HA, LH-HA and BB-A) were activated as follows: 3 g support was incubated with 12 ml 0.1 M potassium phosphate buffer (pH 8.0), which was stirred (200 rpm) in an orbital shaker for 1 h and the pH was maintained between 7.8–8.2. Then, the support was filtered and added to 12 ml 2% (w/v) glutaraldehyde in 0.02 M potassium phosphate buffer (pH 8.0), and stirred (200 rpm) at 25°C for 1 h. The activated support was thoroughly rinsed with deionized water and stored at 4°C (used within 24 h).

#### 2.9.2. Preparation and activation of chitosan beads

Chitosan beads were prepared according to the reported methods with some modification [30], [31]. 2 g of chitosan powder was added to 200 ml of 1.5% (v/v) acetic acid solution (70–80°C). The obtained 1% (v/v) chitosan solution was dropped into a gently stirred 1 M NaOH 30% (v/v) methanol solution through a syringe at room temperature. The beads of dia. 2.5–3.0 mm with uniform shape were selected and immediately washed with plenty of deionized water until the solution became neutral, and then stored in water at 4°C till activation.

10 g chitosan beads were added to 40 ml 2% (w/v) glutaraldehyde in 0.02 M potassium phosphate buffer (pH 8.0) and stirred (200 rpm) in an orbital shaker at 25°C for 5 h. The activated beads were thoroughly rinsed with deionized water and stored in water at 4°C (used within 24 h).

#### 2.9.3 Immobilization of papain onto activated supports

Generally, support of different weights were added to 5 ml enzyme solution (the PEG phase from ATPS) in 25 ml screw-capped glass vial, and the mixture was stirred at 25°C and 200 rpm in an orbital shaker. The protein concentration of the supernatant was determined at intervals. The immobilized enzyme particles were first washed with deionized water, then rinsed with 1 M NaCl solution (prepared with 0.02 M pH 7.0 potassium phosphate buffer), and finally washed thoroughly with 0.02 M pH 7.0 potassium phosphate buffer. The immobilized enzymes were taken to assay their activities and stored at 4°C. The immobilization yield and activity recovery were calculated as follows:
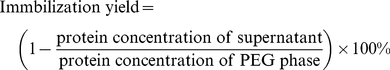
(1)

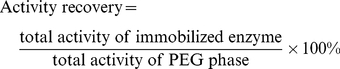
(2)


#### 2.9.4. Preparation of cross-linked papain aggregates from PEG phase

CLEAs of papain were prepared according to the reported method [32]. Precipitant screening: 450 µl of precipitant (acetone, acetonitrile, DMSO, dioxane, ethanol, propanol, iso-propanol, butanol, pentanol, hexanol) was added to 50 µl enzyme solution (the PEG phase from the ATPS). The precipitation was allowed to last for 15 min at 4°C. Then, the mixture was centrifuged (12,000 rpm, Eppendorf 5415D) and the precipitates were redissolved in 500 µl activity buffer. The activity of redissolved precipitates was measured. The appropriate ratio of precipitant to enzyme solution was also investigated.

CLEAs preparation: The pilot assays yielded optimal enzyme precipitation when propanol was used at precipitant/enzyme solution ratio 4∶1. So, 0.8 ml propanol was added to 0.2 ml enzyme solution. The mixture was allowed to precipitate for 15 min at 4°C. Then, appropriate amount of glutaraldehyde solution (25%, w/v) was added into the suspensions to attain the desired concentration (0.2%, 0.5%, 1%, 2%), and the mixture was stirred at 25°C and 200 rpm for 2 h. After cross-linking, the cross-linked aggregates were quenched with 9-fold volume of activity buffer. A sample (A) containing CLEAs as well as residual free enzyme was withdrawn from the suspension and assayed for activity. Then, the CLEAs were centrifuged off (20,000×g, 15 min), and the supernatant containing only free enzyme was withdrawn as a sample (B). The difference in activity between sample A and B was the CLEAs activity.

The pilot assays yielded optimal active CLEAs when propanol was used at a ratio of 4/1 with a 2 h cross-linking period at 0.5% glutaraldehyde. To scale-up the CLEAs production, an initial 20 ml enzyme solution was used. At the end of the cross-linking period, the entire suspension was centrifuged at 20,000×g and 4°C for 15 min. The precipitated CLEAs collected were washed three times with deionized water. Finally, the preparation of the CLEAs was lyophilized to obtain dried powder.

## Results and Discussion

### 3.1. Plackett-Burman screening

According to the earlier published reports [7], [12] and our preliminary tests, seven factors were considered to perform the P-B design (Table 1). According to the experimental data analysis (taking the best papain purity and activity recovery into account), three variables namely PEG concentration, salt concentration and pH had significant effect (data not shown). The ATPS was preferred at initial protein concentration 15 mg/ml, PEG 6000, NaH_2_PO_4_/K_2_HPO_4_ and 20°C.

### 3.2. Optimization of screened factors

Central composite design (CCD) was employed to optimize the three most significant factors (PEG concentration (C_PEG_, %), salt concentration (C_salt_, %) and pH) identified by P-B design for enhancing the responses of papain purity (P_PAP_, %) and total activity of PEG phase (A_TOP_, nkat). The three variables were studied at five levels and a set of 20 experiments was carried out (Table 2).

The responses of P_PAP_ and A_TOP_ could be best fitted using second-order polynomial equation as follows:
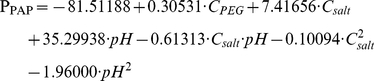
(3)

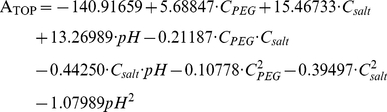
(4)


Both the models were verified using ANOVA (see Supplementary Table S1 and S2). The regression model was determined by the Design Expert procedure that considered initially all the factors and then eliminated those having no effect step-by-step. The significance of each term in the model was evaluated by its corresponding P value. The value less than 0.05 indicated that the terms were significant, whereas the value more than 0.1 indicated that the terms were not significant. The large F value (45.56 for P_PAP_ and 89.11 for A_TOP_) and very low P value (<0.0001 for both P_PAP_ and A_TOP_) suggested that both models were significant at high confidence level. The lack of fit values (1.22 for P_PAP_ and 3.68 for A_TOP_) were not significant with respect to their corresponding pure error, which proved that both models could be fitted to evaluate the responses. Furthermore, the fitness of the models was assessed by determination coefficient (R^2^). Adjusted R^2^ (0.93 for P_PAP_ and 0.97 for A_TOP_), suggesting more than 90% of the variation due to the variables presented in the models, were in reasonable agreement with the predicted R^2^ (0.87 for P_PAP_ and 0.92 for A_TOP_). High R^2^ (0.95 for P_PAP_ and 0.98 for A_TOP_) indicated a good agreement between predicted and experimental values.

**Figure 1:**
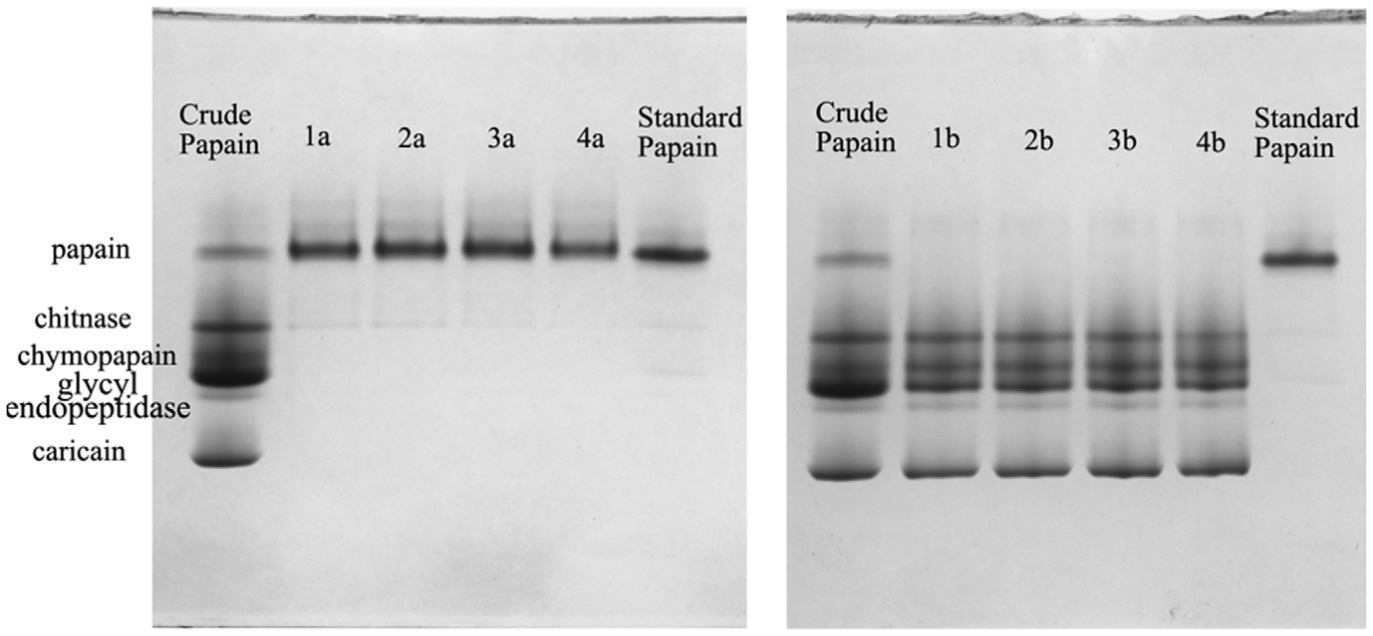
Native-PAGE of papain during extraction in ATPS: 1a-4a corresponding to the run number of Table 3 represented the PEG phase of ATPS; 1b-4b corresponding to the run number of Table 3 represented the salt phase of ATPS; Crude papain: spray-dried latex powders; Standard papain: 2×crystallized papain. All the samples were loaded 10 µg.

The criterion for the numerical solution was evaluated by setting the maximum goals for P_PAP_ and A_TOP_ with different importance values while the other variables were in their range. The predicted solutions and the experimental results were shown in Table 3 (Run 1–4). The higher purity of papain was obtained, the lower total activity of PEG phase was observed. The results presented in Table 3 (Run 1–4) clearly indicated optimization was effective for purifying papain using ATPS. The Native-PAGE (Figure 1) also confirmed that papain was extracted to the PEG phase. The proteins (often called as crude papain) in the spray-dried latex powders were separated into five bands on the electrophoresis. One of these proteins was identified as papain according to the standard papain and other protein bands were identified by the published reports [6], [26], [33]. The electrophoresis patterns indicated that the purity of obtained papain was improved with the increase of C_PEG_, C_salt_ and pH (increased the importance value of P_PAP_), and all the purified papain was purer than the commercially available purest one obtained by 2×crystallized.

**Table 3:** Constraints targeting for both P_PAP_ and A_TOP_ and its solutions according to the model.

Run	A:C_PEG_ %	B:C_salt_ %	C:pH	P_PAP_ %			A_TOP_ nkat			Desirability
				Predicted	Experimental	Importance	Predicted	Experimental	Importance	
1	14.33	14.27	5.77	96.08	95.24±0.1	3	11.52	10.71±0.1	3	0.78
2	15.10	14.30	5.98	97.09	96.72±0.4	5	10.53	9.64±0.1	3	0.79
3	15.89	14.35	6.13	97.78	97.94±1.0	5	9.64	9.18±0.1	2	0.80
4	17.63	14.42	6.30	98.73	98.72±0.2	5	7.89	7.83±0.1	1	0.84
5	14.33	14.27	5.77	96.08	96.44±0.1	3	230.38	247.50±2.5	3	0.78
6	17.63	14.42	6.30	98.73	100.00±0.1	5	157.79	168.55±1.7	1	0.84

Run 1–5: confirmation experiments of small scale (10 g) ATPS; confirmation experiments of large scale (200 g) ATPS.

To confirm whether the optimum operating conditions established for the PEG/phosphate system could indeed provide desired outcome in large scale, validation experiments (Table 3, Run 5–6) were performed using 200 g ATPS, in which consistent results were yielded comparable to those obtained in a smaller system (10 g). Therefore the optimum operating conditions for purifying papain in ATPS could be concluded as: 40% (w/w) 15 mg/ml enzyme solution, 14.33–17.65% (w/w) PEG 6000, 14.27–14.42% (w/w) NaH2PO4/K2HPO4 and pH 5.77–6.30 at 20°C. The purity of papain obtained ranged from 96% to 100%.

### 3.3. In situ immobilization of papain from PEG phase on aminated supports

The immobilization of enzymes on glutaraldehyde preactivated supports is quite simple and efficient, and in some instances even improves the enzyme stability by multipoint or multisubunit immobilization. In general, the immobilization of enzyme on preactivated aminated supports follows a two-step mechanism: firstly, a rapid modest ionic exchange absorption of the enzyme occurs on the support; and secondly the covalent reaction between the absorbed enzyme and activated groups on the support takes place [34]. So it is important to know when the enzyme is absorbed onto the support and when the immobilization is finished, i.e. make clear the immobilization course.

**Figure 2:**
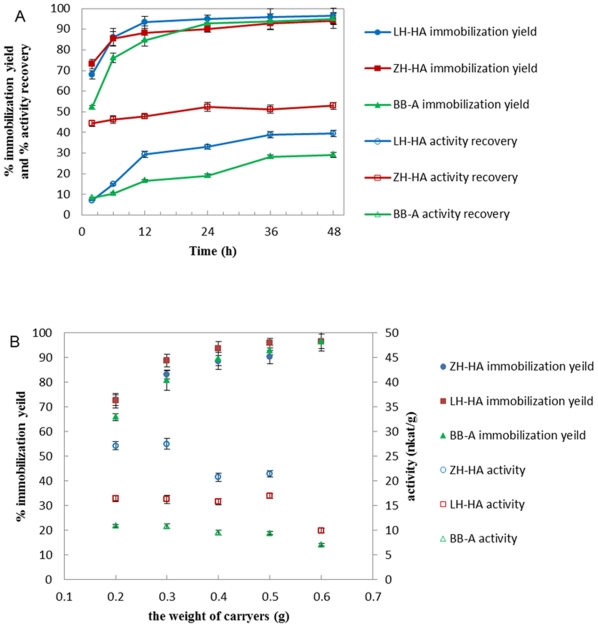
In situ immobilization of papain on aminated supports. (A) The immobilization course: the experiment was conducted by incubating 0.5 g supports in 5 ml enzyme solutions at 25°C, 200 rpm. (B) Effect of supports input amount on immobilization: the experiment was conducted by incubating different weight of supports in 5 ml enzyme solutions at 25°C, 200 rpm.

Figure 2A presented the variation of immobilization yield and activity recovery of papain versus immobilization time. In the first 12 h, papain was quickly absorbed onto the surface of the supports and the activity recovery increased rapidly. After 12 h, the immobilization yield and activity recovery slowed down because proteins slowly diffused into the porus of supports and reacted with the inside activated groups. The immobilization on ZH-HA finished at 24 h and the immobilization yield of 90.2% and activity recovery of 52.5% were achieved. The immobilization on LH-HA and BB-A finished at 36 h and the immobilization yields (more than 90%) of these two supports were almost the same as ZH-HA, but the activity recovery was only 38.9% and 28.2%, respectively. The appropriate support/enzyme solution ratio (g/ml) was also investigated. The results showed that the best ratios for ZH-HA, LH-HA and BB-A were 0.3/5, 0.5/5 and 0.5/5, respectively (Figure 2B).

### 3.4. In situ immobilization of papain from PEG phase on chitosan beads

**Figure 3:**
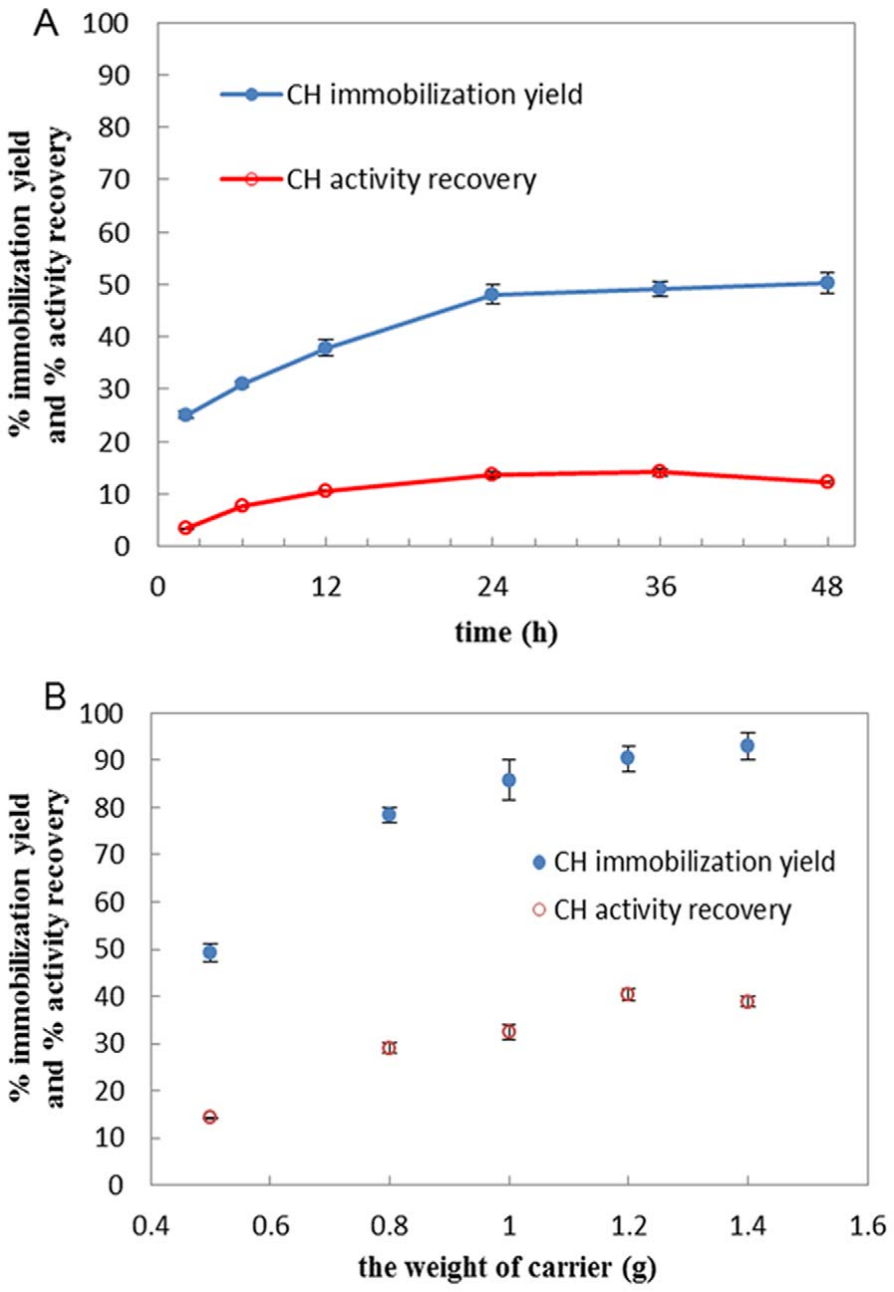
In situ immobilization of papain on chitosan beads. (A) The immobilization course: the experiment was conducted by incubating 0.5 g chitosan beads in 5 ml enzyme solutions at 25°C, 200 rpm. (B) Effect of chitosan beads input amount on immobilization: the experiment was conducted by incubating different weight of chitosan beads in 5 ml enzyme solutions at 25°C, 200 rpm.

The mechanism of immobilizing papain onto chitosan beads (CH) is similar to that of aminated support. The immobilization yield and activity recovery of papain versus immobilization time was presented in Figure 3A. As shown, the immobilization on chitosan beads finished at 36 h and by when the maximum immobilization yield and activity recovery were achieved. The optimal ratio of chitosan beads to enzyme solution was further investigated as shown in Figure 3B, i.e. 1.2/5 (g/ml), in which the immobilization yield and the activity recovery reached to 90.4% and 40.3% respectively.

In this work, we also tested the in situ immobilization of papain on epoxy supports such as Eupergit C, Amerzyme and LH-EP. Unfortunately, the immobilization yield and activity recovery for all the epoxy supports were very low (data not shown). There may be three reasons for this phenomenon: (1) The low ionic strength of the PEG phase could not promote the enzyme to absorb onto the high hydrophobic surface of the epoxy supports; (2) The pH of the PEG phase was acid, but the covalent reaction between the absorbed enzyme and activated groups on supports was promoted at alkaline pH; (3) The epoxy groups on supports might react with the thiol group inside the active site of papain, and thus inactivated papain [35].

In situ immobilization of papain from PEG phase not only realized the separation of papain from PEG and avoided the use of other purification steps, but also opened a door for reusing the phase-forming polymer (PEG). After in situ immobilization and filtering out the supports, the top phase mixed with the bottom phase portion to reform the ATPS which could be used for further purification of papain [17].

### 3.5. Preparation of CLEAs from PEG phase

CLEAs preparation consists of two steps: aggregation by precipitation and cross-linking. Precipitation by the addition of salts, organic solvents or nonionic polymers to the enzyme solutions, is a commonly used method for enzyme purification [32]. The resulting physical aggregates of enzyme molecules are supramolecular structures that are held together by non-covalent bonding and can be easily redissolved in water. Cross-linking produces insoluble CLEAs in which the structural properties and catalytic activities of the enzyme are maintained. Due to the different biochemical and structural properties of enzymes, the best precipitant and cross-linker can vary from one enzyme to another [36].

**Figure 4:**
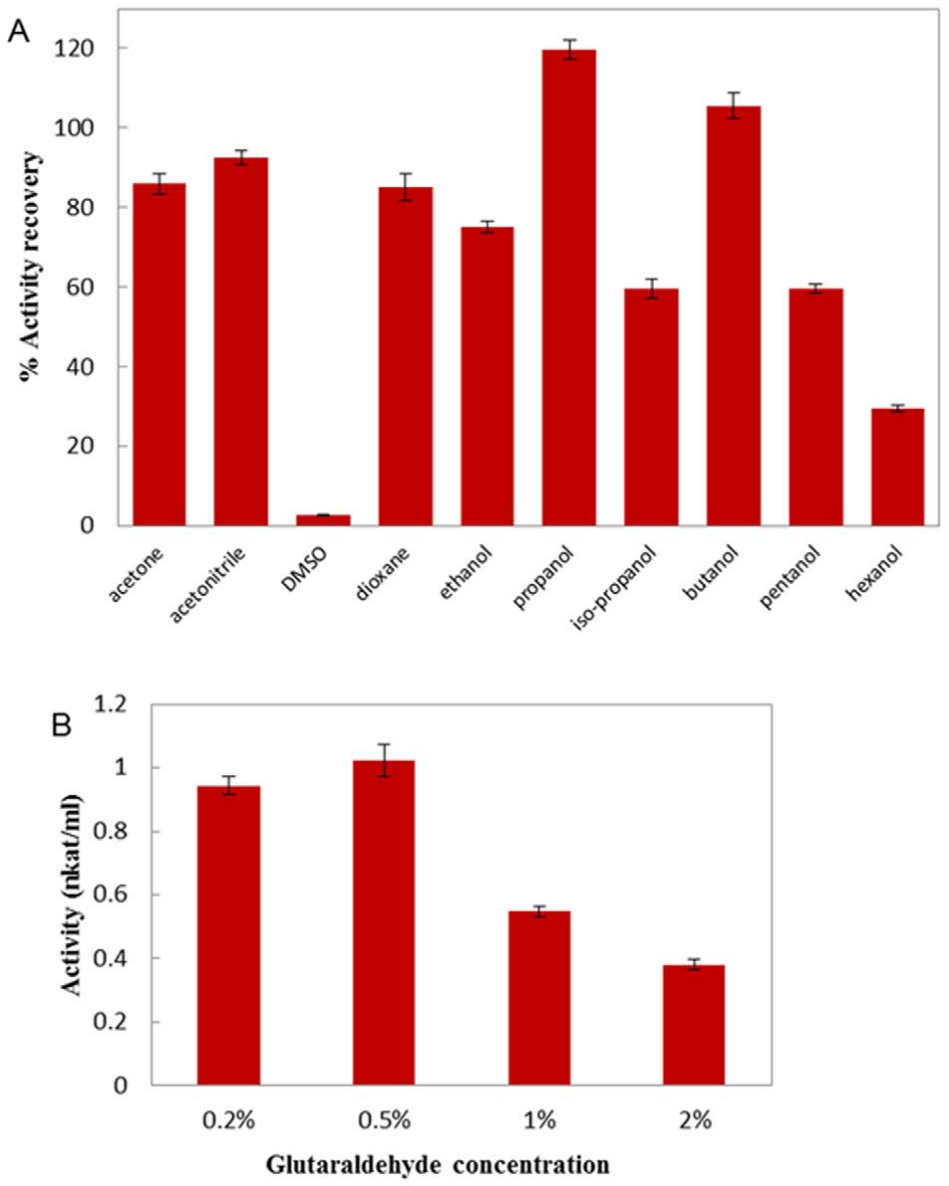
Preparation of CLEAs from PEG phase. (A) Precipitant screen. (B) Glutaraldehyde concentration screen.

Our work was carried out by precipitating the purified papain from PEG phase followed by cross-linking the aggregates using glutaraldehyde. In the screening of precipitants, propanol was found to generate solid enzyme aggregates with almost 120% activity upon resolubilization through dilution of the precipitant (Figure 4A). This hyperactivation was thought to find its origin in conformational changes of the protein induced by the aggregated state [37]. Similar phenomenon was also observed by R. Schoevaart et al. [32]. We also found the optimal ratio of propanol to enzyme solution for completely precipitating papain was 4/1 (v/v).

R. Schoevaart et al. reported that temperature had little effect on precipitation, and generally at room temperature there was no increase in cross-linking observed after 3 h [32]. So we carried out cross-linking at 25°C, and the reaction was quenched after 2 h. Glutaraldehyde was usually chosen as the cross-linker as it was inexpensive and readily available in quantities. In preparing the CLEAs, the concentration of glutaraldehyde should be optimized. If too little cross-linker was used, the enzyme molecule might still be too flexible. Whereas too much cross-linker could result in a loss of the minimum flexibility needed for the activity of enzyme [36]. Figure 4B presented the CLEAs activity after cross-linking at different glutaraldehyde concentrations. As shown, the CLEAs obtained the maximum activity at 0.5% glutaraldehyde.

**Figure 5:**
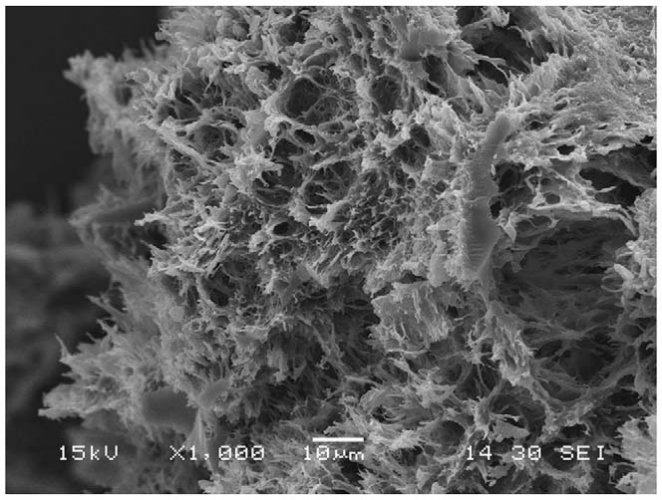
Scanning electron microscope images of lyophilized CLEAs. After gold sputtering, the samples were visualized with a JSM-6360LV Electron Microscope (JEOL) operated at 15 kV.

To test the validity of the parameters found in the small-scale pilot assays of CLEAs preparations, we scaled up the procedure to a 100-fold. The final product of the CLEAs was lyophilized to get the dry powder. The dried CLEAs have a hydrolytic activity hundreds of times higher than those of carrier-bound immobilized papain (360.0 nkat/g for CLEAs, 27.5 nkat/g for ZH-HA, 16.9 nkat/g for LH-HA, 10.9 nkat/g for BB-A, 5.0 nkat/g for CH). This is because that a distinct disadvantage of carrier-bound enzymes, whether they involve binding to or encapsulation in a carrier, is the dilution of catalytic activity resulting from the introduction of a large proportion of noncatalytic mass, generally ranging from 90 to >99% of the total mass. This inevitably leads to lower volumetric, space-time yields and lower catalyst productivities. However, CLEAs do not suffer from this disadvantage, because the molecular weight of the cross-linker is negligible compared with that of the enzyme [38]. These were also confirmed by the scanning electron microscopy of CLEAs papain (Fig. 5). As shown, the CLEAs had large open channels and loose structures, which could overcome the diffusion limitation often observed in carrier-bound immobilization [20], [39].

### Conclusions

The feasibility of using ATPS for the purification of papain from the spray-dried papaya latex followed by enzyme immobilization was shown in this paper. RSM was used to optimize the ATPS process. The optimum process conditions were 40% (w/w) 15 mg/ml enzyme solution, 14.33–17.65% (w/w) PEG 6000, 14.27–14.42% (w/w) NaH2PO4/K2HPO4 and pH 5.77–6.30 at 20°C. The purity of papain could attain to 96–100%. In situ immobilization of papain in the PEG phase resulted in very high immobilization yield (>90% for all supports except for ZH-HA) and better activity recovery (43.3% for ZH-HA, 38.9% for LH-HA, 28.2% for BB-A and 40.3% for CH). Moreover, preparation of CLEAs was realized to recover papain from PEG phase for the first time and the obtained CLEAs had a hydrolytic activity hundreds of times higher than those of carrier-bound immobilized papain.

## Supporting Information

Table S1 ANOVA for papain purity in CCD.
(DOC)

Table S2 ANOVA for total activity of PEG phase in CCD.
(DOC)

## References

[1] Azarkan M, El Moussaoui A, van Wuytswinkel D, Dehon G, Looze Y (2003). Fractionation and purification of the enzymes stored in the latex of *Carica papaya*.. Journal of Chromatography B.

[2] Walsh G (2002). Proteins: Biochemistry and Biotechnology..

[3] Morcelle SR, Barberis S, Priolo N, Caffini NO, Clapés P (2006). Comparative behaviour of proteinases from the latex of *Carica papaya* and *Funastrum clausum* as catalysts for the synthesis of Z-Ala-Phe-OMe.. Journal of Molecular Catalysis B: Enzymatic.

[4] Baines BS, Brocklehurst K (1979). A necessary modification to the preparation of papain from any high-quality latex of *Carica papaya* and evidence for the structural integrity of the enzyme produced by traditional methods.. Biochem J.

[5] Kimmel JR, Smith EL (1954). Crystalline papain. I. preparation, specificity, and activation.. J Biol Chem.

[6] Monti R, Basilio CA, Trevisan HC, Contiero J (2000). Purification of papain from fresh latex of *Carica papaya*.. Brazilian Archives of Biology and Technology.

[7] Nitsawang S, Hatti-Kaul R, Kanasawuda P (2006). Purification of papain from *Carica papaya* latex: Aqueous two-phase extraction versus two-step salt precipitation.. Enzyme and Microbial Technology.

[8] Rito-Palomares M (2004). Practical application of aqueous two-phase partition to process development for the recovery of biological products.. Journal of Chromatography B.

[9] Forciniti D, Hatti-Kaul R (2000). Preparation of Aqueous Two-Phase Systems.. Aqueous Two-Phase Systems: Methods and Protocols.

[10] Selber K, Tjerneld F, Collen A, Hyytia T, Nakari-Setala T (2004). Large-scale separation and production of engineered proteins, designed for facilitated recovery in detergent-based aqueous two-phase extraction systems.. Process Biochemistry.

[11] Naganagouda K, Mulimani VH (2008). Aqueous two-phase extraction (ATPE): An attractive and economically viable technology for downstream processing of Aspergillus oryzae α-galactosidase.. Process Biochemistry.

[12] Kuboi R, Wang WH, Komasawa I (1990). Effect of contaminating proteins on the separation and purification of papain from papaya latex using aqueous two-phase extraction.. Kagaku Kogaku Ronbunshu.

[13] Menge U, Hatti-Kaul R (2000). Optimization of Extractions in Aqueous Two-Phase Systems.. Aqueous Two-Phase Systems: Methods and Protocols.

[14] Ashipala OK, He Q (2008). Optimization of fibrinolytic enzyme production by *Bacillus subtilis* DC-2 in aqueous two-phase system (poly-ethylene glycol 4000 and sodium sulfate).. Bioresource Technology.

[15] Kammoun R, Chouayekh H, Abid H, Naili B, Bejar S (2009). Purification of CBS 819.72 α-amylase by aqueous two-phase systems: Modelling using Response Surface Methodology.. Biochemical Engineering Journal.

[16] Johansson G, Hatti-Kaul R (2000). Recovery of Proteins and Phase Components.. Aqueous Two-Phase Systems: Methods and Protocols.

[17] Guan YH, Lilley TH, Brook AH (2001). Production of immobilized penicillin acylase using aqueous polymer systems for enzyme purification and in situ immobilization.. Enzyme and Microbial Technology.

[18] Bradoo S, Saxena RK, Gupta R (1999). Partitioning and resolution of mixture of two lipases from *Bacillus stearothermophilus* SB-1 in aqueous two-phase system.. Process Biochemistry.

[19] Zhang Y-y, Liu J-h (2010). Purification and in situ immobilization of lipase from of a mutant of *Trichosporon laibacchii* using aqueous two-phase systems.. Journal of Chromatography B.

[20] Kallenberg AI, Rantwijk Fv, Sheldon RA (2005). Immobilization of Penicillin G Acylase: The Key to Optimum Performance.. Advanced Synthesis & Catalysis.

[21] Rosa PAJ, Azevedo AM, Aires-Barros MR (2007). Application of central composite design to the optimisation of aqueous two-phase extraction of human antibodies.. Journal of Chromatography A.

[22] Albertsson PA (1986). Partition of cell particles and macromolecules..

[23] Bradford M (1976). A rapid and sensitive method for the quantitation of microgram quantities of protein utilizing the principle of protein-dye binding.. Analytical Biochemistry.

[24] Azarkan M, Dibiani R, Baulard C, Baeyens-Volant D (2006). Effects of mechanical wounding on *Carica papaya* cysteine endopeptidases accumulation and activity.. International Journal of Biological Macromolecules.

[25] Dekeyser PM, De Smedt S, Demeester J, Lauwers A (1994). Fractionation and purification of the thiol proteinases from papaya latex.. Journal of Chromatography B: Biomedical Sciences and Applications.

[26] Dubois T, Jacquet A, Schnek AG, Looze Y (1988). The thiol proteinases from the latex of *Carica papaya* L. I. Fractionation, purification and preliminary characterization.. Biol Chem Hoppe Seyler.

[27] Erlanger BF, Kokowsky N, Cohen W (1961). The preparation and properties of two new chromogenic substrates of trypsin.. Archives of Biochemistry and Biophysics.

[28] Su SN, Nie HL, Zhu LM, Chen TX (2009). Optimization of adsorption conditions of papain on dye affinity membrane using response surface methodology.. Bioresource Technology.

[29] Reisfeld RA, Lewis UJ, Williams DE (1962). Disk Electrophoresis of Basic Proteins and Peptides on Polyacrylamide Gels.. Nature.

[30] Kumar S, Dwevedi A, Kayastha AM (2009). Immobilization of soybean (Glycine max) urease on alginate and chitosan beads showing improved stability: Analytical applications.. Journal of Molecular Catalysis B: Enzymatic.

[31] Bhandari S, Gupta VK, Singh H (2009). Enhanced stabilization of mungbean thiol protease immobilized on glutaraldehyde-activated chitosan beads.. Biocatalysis and Biotransformation.

[32] Schoevaart R, Wolbers MW, Golubovic M, Ottens M, Kieboom APG (2004). Preparation, optimization, and structures of cross-linked enzyme aggregates (CLEAs).. Biotechnology and Bioengineering.

[33] Chaiwut P, Nitsawang S, Shank L, Kanasawud P (2007). A Comparative Study on Properties and Proteolytic Components of Papaya Peel and Latex Proteases.. Chiang Mai J Sci.

[34] Betancor L, López-Gallego F, Alonso-Morales N, Dellamora G, Mateo C, Guisan JM (2006). Glutaraldehyde in Protein Immobilization.. Immobilization of Enzymes and Cells.

[35] Mateo C, Abian O, Fernández-Lorente G, Pessela BCC, Grazu V, Guisan JM (2006). Immobilization-Stabilization of Enzymes by Multipoint Covalent Attachment on Supports Activated With Epoxy Groups.. Immobilization of Enzymes and Cells.

[36] Matijosyte I, Arends IWCE, de Vries S, Sheldon RA (2010). Preparation and use of cross-linked enzyme aggregates (CLEAs) of laccases.. Journal of Molecular Catalysis B: Enzymatic.

[37] López-Serrano P, Cao L, van Rantwijk F, Sheldon RA (2002). Cross-linked enzyme aggregates with enhanced activity: application to lipases.. Biotechnology Letters.

[38] Sheldon RA, Schoevaart R, Langen LM, Guisan JM (2006). Cross-Linked Enzyme Aggregates.. Immobilization of Enzymes and Cells.

[39] Cao L, van Rantwijk F, Sheldon RA (2000). Cross-Linked Enzyme Aggregates: A Simple and Effective Method for the Immobilization of Penicillin Acylase.. Organic Letters.

