# Supplementary material for: Purification and In Situ Immobilization of Papain with Aqueous Two-Phase System
Source: PLoS One. 2010 Dec 13;5(12):e15168. doi: 10.1371/journal.pone.0015168 (PMC3001450; doi:10.1371/journal.pone.0015168)
Supplement: Table S1 — ANOVA for papain purity in CCD. (DOC) [file pone.0015168.s001.doc]

**Table S1**

ANOVA for papain purity in CCD.

| Source | Sum of squares | df | Mean square | F value | p-value Prob>F | Significant term based on Prob>F value |
| --- | --- | --- | --- | --- | --- | --- |
| Model | 335.90 | 6 | 55.98 | 45.56 | < 0.0001 | significant |
| A-PEG | 5.97 | 1 | 5.97 | 4.86 | 0.0462 | significant |
| B-Salt | 53.18 | 1 | 53.18 | 43.28 | < 0.0001 | significant |
| C-pH | 163.39 | 1 | 163.39 | 132.98 | < 0.0001 | significant |
| BC | 12.03 | 1 | 12.03 | 9.79 | 0.0080 | significant |
| B2 | 4.30 | 1 | 4.30 | 3.50 | 0.0842 |  |
| C2 | 101.24 | 1 | 101.24 | 82.40 | < 0.0001 | significant |
| Residual | 15.97 | 13 | 1.23 |  |  |  |
| Lack of Fit | 10.57 | 8 | 1.32 | 1.22 | 0.4306 | not significant |
| Pure Error | 5.40 | 5 | 1.08 |  |  |  |
| Cor Total | 351.87 | 19 |  |  |  |  |
| std. dev. | 1.11 |  | R-squared | | 0.95 |  |
| Mean | 95.26 |  | Adj R-squared | | 0.93 |  |
| C.V. | 1.16 |  | Pred R-squared | | 0.87 |  |
| Press | 45.08 |  | Adeq precision | | 24.81 |  |
