# Supplementary material for: Purification and In Situ Immobilization of Papain with Aqueous Two-Phase System
Source: PLoS One. 2010 Dec 13;5(12):e15168. doi: 10.1371/journal.pone.0015168 (PMC3001450; doi:10.1371/journal.pone.0015168)
Supplement: Table S2 — ANOVA for total activity of PEG phase in CCD. (DOC) [file pone.0015168.s002.doc]

**Table S2**

ANOVA for total activity of PEG phase in CCD.

| Source | Sum of squares | df | Mean square | F value | p-value Prob>F | Significant term based on Prob>F value |
| --- | --- | --- | --- | --- | --- | --- |
| Model | 848.48 | 8 | 106.06 | 89.11 | < 0.0001 | significant |
| A-PEG | 33.81 | 1 | 33.81 | 28.41 | 0.0002 | significant |
| B-Salt | 171.48 | 1 | 171.48 | 144.08 | < 0.0001 | significant |
| C-pH | 553.90 | 1 | 553.90 | 465.39 | < 0.0001 | significant |
| AB | 5.75 | 1 | 5.75 | 4.83 | 0.0503 |  |
| BC | 6.27 | 1 | 6.27 | 5.26 | 0.0424 | significant |
| A2 | 4.67 | 1 | 4.67 | 3.93 | 0.0731 |  |
| B2 | 62.67 | 1 | 62.76 | 52.73 | < 0.0001 | significant |
| C2 | 29.32 | 1 | 29.32 | 24.64 | 0.0004 | significant |
| Residual | 13.09 | 11 | 1.19 |  |  |  |
| Lack of Fit | 10.67 | 6 | 1.78 | 3.68 | 0.0871 | not significant |
| Pure Error | 2.42 | 5 | 0.48 |  |  |  |
| Cor Total | 861.57 | 19 |  |  |  |  |
| std. dev. | 1.09 |  | R-squared | | 0.98 |  |
| Mean | 15.28 |  | Adj R-squared | | 0.97 |  |
| C.V. | 7.14 |  | Pred R-squared | | 0.92 |  |
| Press | 70.10 |  | Adeq precision | | 32.16 |  |
